# O.5.2-7 Listening to the patients’ voice: a conceptual framework of the walking experience

**DOI:** 10.1093/eurpub/ckad133.248

**Published:** 2023-09-11

**Authors:** Laura Delgado-Ortiz, Ashley Polhemus, Alison Keogh, Norman Sutton, Werner Remmele, Clint Hansen, Felix Kluge, Basil Sharrack, Clemens Becker, Thierry Troosters, Walter Maetzler, Lynn Rochester, Anja Frei, Milo Puhan, Judith Garcia-Aymerich

**Affiliations:** Non-Communicable Diseases and Environment Programme, ISGlobal, Barcelona, Spain; Department of Medicine and Life Sciences, Universitat Pompeu Fabra, Barcelona, Spain; CIBER Epidemiología y Salud Pública, Barcelona, Spain; Epidemiology, Biostatistics and Prevention Institute, University of Zurich, Zurich, Switzerland; Insight Centre for Data Analytics, University College Dublin, Dublin, Ireland; Mobilise-D Patient and Public Advisory Group; Mobilise-D Patient and Public Advisory Group; Department of Neurology, University Medical Center Schleswig-Holstein, Kiel, Germany; Department of Artificial Intelligence in Biomedical Engineering, Friedrich-Alexander- Universität Erlangen-Nürnberg (FAU), Erlangen, Germany; Department of Neuroscience and Sheffield NIHR Translational Neuroscience BRC, Sheffield Teaching Hospitals NHS Foundation Trust & University of Sheffield, Sheffield, UK; Department of Clinical Gerontology, Robert-Bosch-Hospital, Stuttgart, Germany; Department of Rehabilitation Sciences, KU Leuven, Leuven, Belgium; Department of Respiratory Diseases, University Hospitals Leuven, Leuven, Belgium; Translational and Clinical Research Institute, Faculty of Medical Sciences, Newcastle University, Newcastle upon Tyne, UK; laura.delgado@isglobal.org; Epidemiology, Biostatistics and Prevention Institute, University of Zurich, Zurich, Switzerland; Epidemiology, Biostatistics and Prevention Institute, University of Zurich, Zurich, Switzerland; Non-Communicable Diseases and Environment Programme, ISGlobal, Barcelona, Spain; Department of Medicine and Life Sciences, Universitat Pompeu Fabra, Barcelona, Spain; CIBER Epidemiología y Salud Pública, Barcelona, Spain

## Abstract

**Purpose:**

Walking is crucial for an active and healthy ageing, but it changes with age and in the presence of diverse health conditions, such as non-communicable diseases and injuries. So far, conceptual frameworks of walking have not included the impact of these conditions and individuals’ lived experiences on their walking. Thus, we aimed to identify and synthesize evidence describing the walking experience from the perspective of individuals living with highly prevalent walking-impairing conditions of diverse aetiology (i.e., Parkinson’s disease, multiple sclerosis, chronic obstructive pulmonary disease, heart failure, hip fracture, frailty and sarcopenia).

**Methods:**

We conducted a systematic review and meta-ethnography of qualitative evidence, following appropriate guidance.

**Results:**

Out of 2,552 unique records, 117 were deemed eligible for the meta-ethnographic synthesis. We identified seven common themes that explain the experience of walking: (1) becoming aware of the walking experience, (2) the walking experience as a link between individuals’ activities and sense of self, (3) the physical walking experience, (4) the mental and emotional walking experience, (5) the social walking experience, (6) the context of the walking experience, and (7) behavioral and attitudinal adaptations resulting from the walking experience.

**Conclusion:**

We proposed a framework that describes the interplay between these themes, providing a conceptualization of walking that is grounded in the experiences of individuals recovering from a hip fracture or living with other walking-impairing health conditions, and that may be used to set priorities and improve patient centricity in clinical practice, research and public health initiatives.

**Support/Funding Source:**

This work was supported by the Mobilise-D project that has received funding from the Innovative Medicines Initiative 2 Joint Undertaking (JU) under grant agreement No. 820820. This JU receives support from the European Union’s Horizon 2020 research and innovation programme and the European Federation of Pharmaceutical Industries and Associations (EFPIA).

